# Supplementary figures and images for: Enhanced genome editing in human iPSCs with CRISPR-CAS9 by co-targeting ATP1a1
Source: PeerJ. 2020 May 1;8:e9060. doi: 10.7717/peerj.9060 (PMC7197401; doi:10.7717/peerj.9060)

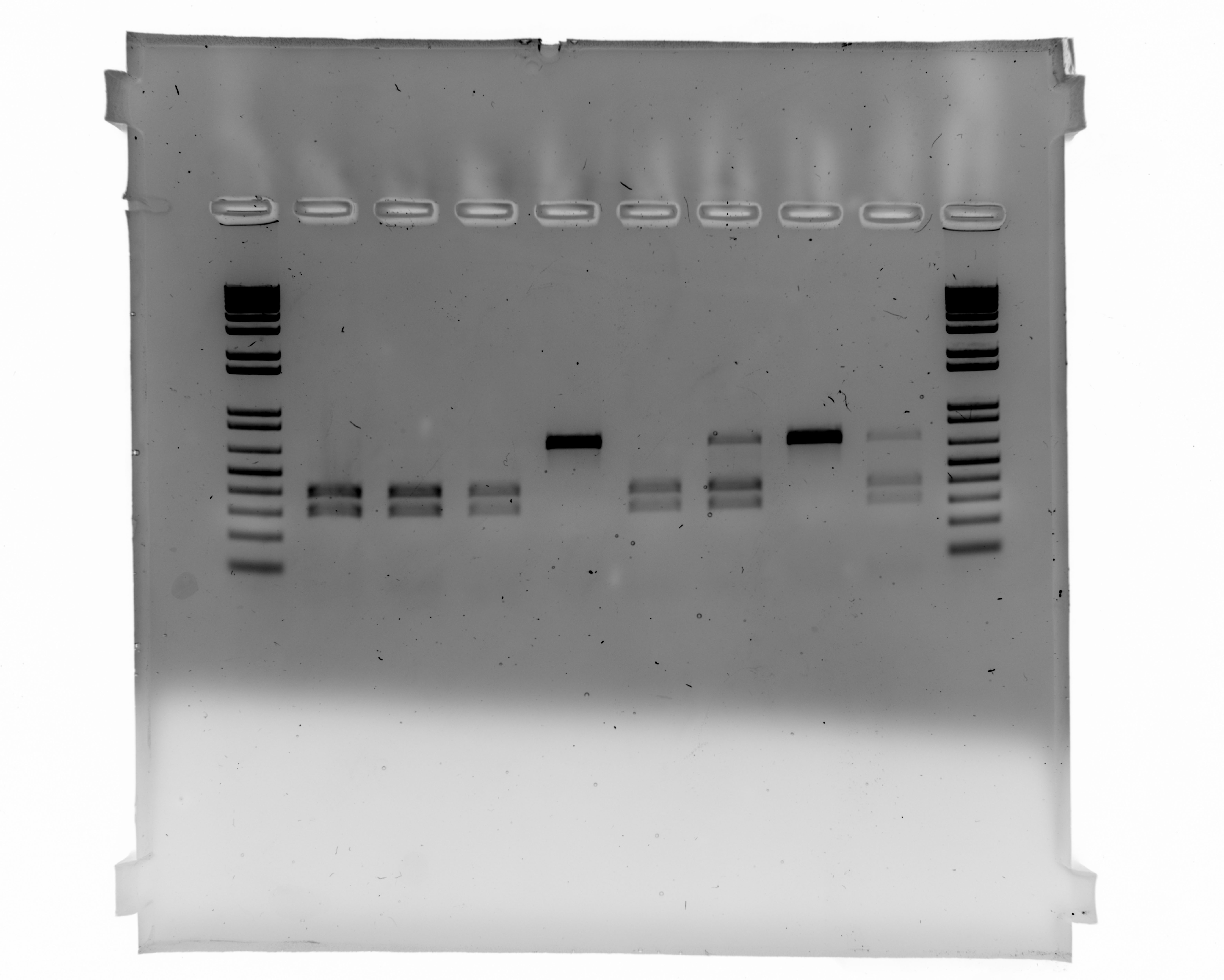

Supplement: Figure S1 — Lane 1:1kb ladder. Lane 6: ATP1A1 118R/129D homozygous; Lane 7: ATP1A1 118R/129D heterozygous; Lane 8: ATP1A1 118Q/129N Wild Type. [file peerj-08-9060-s003.jpg]

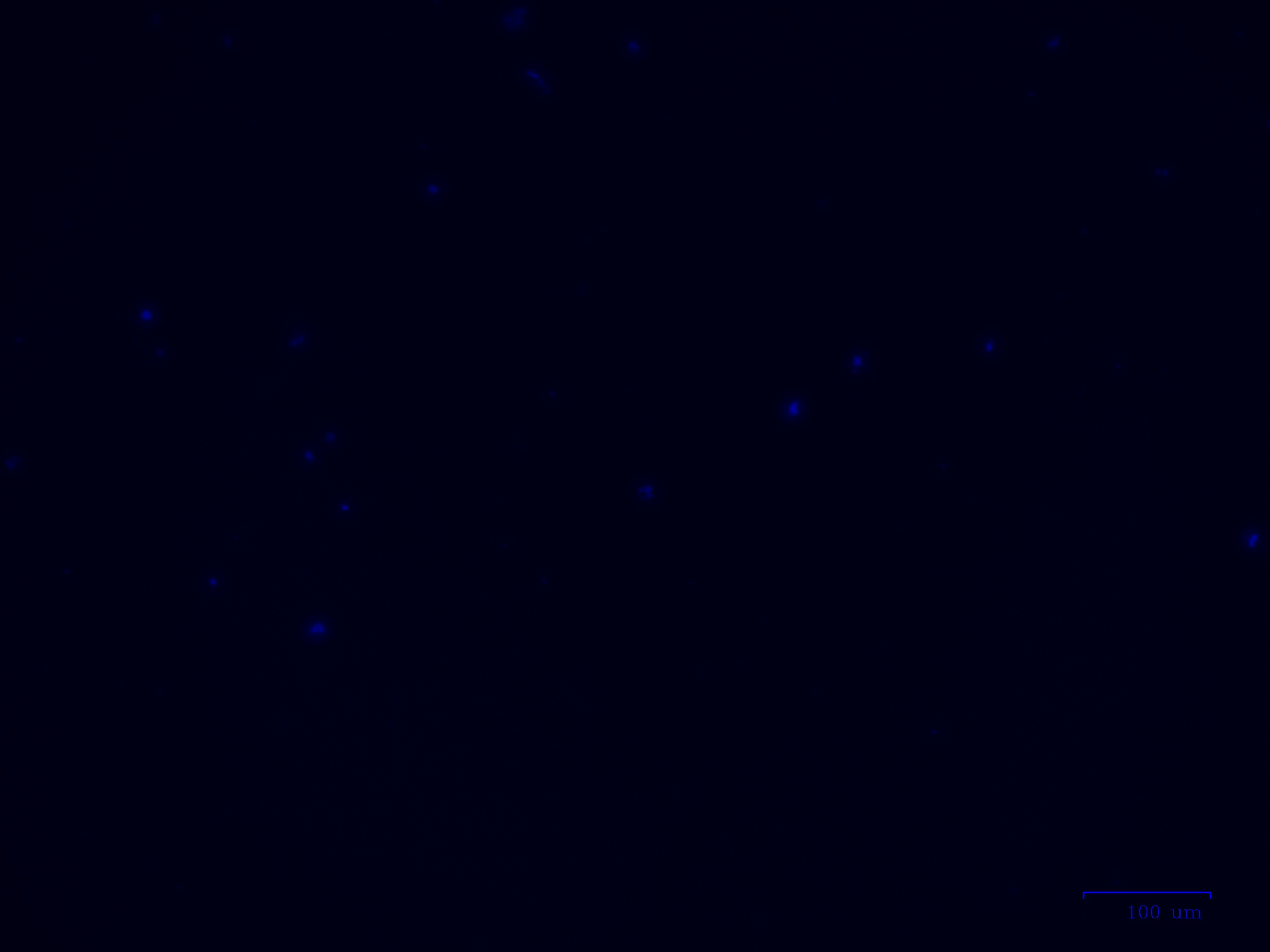

Supplement: Figure S2 — DAPI staining of WT iPSCs treated with 1 uM Ouabain [file peerj-08-9060-s004.jpg]

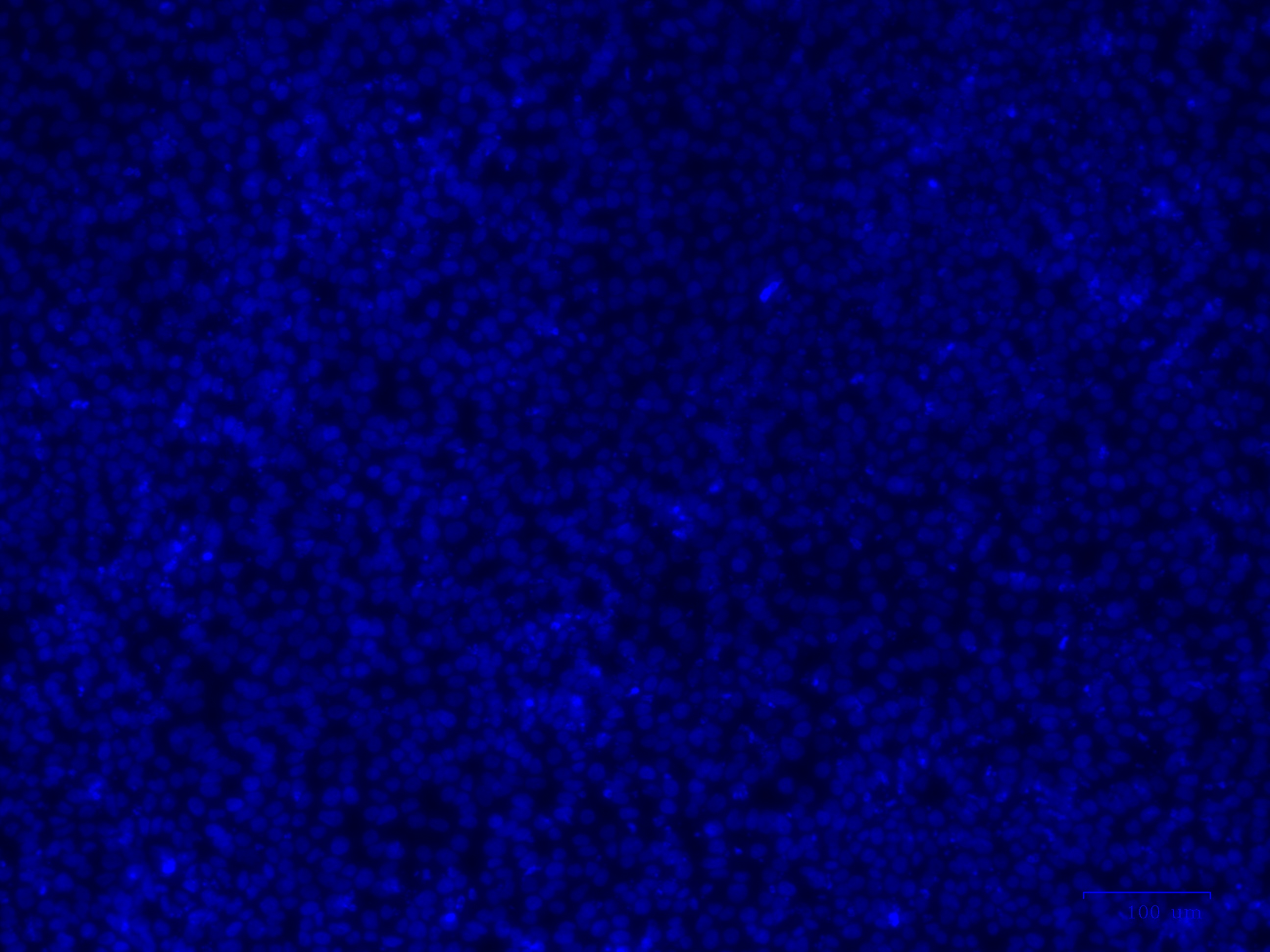

Supplement: Figure S3 — DAPI staining of ATP1A1 (118R/129D) iPSCs treated with 1 uM Ouabain [file peerj-08-9060-s005.jpg]

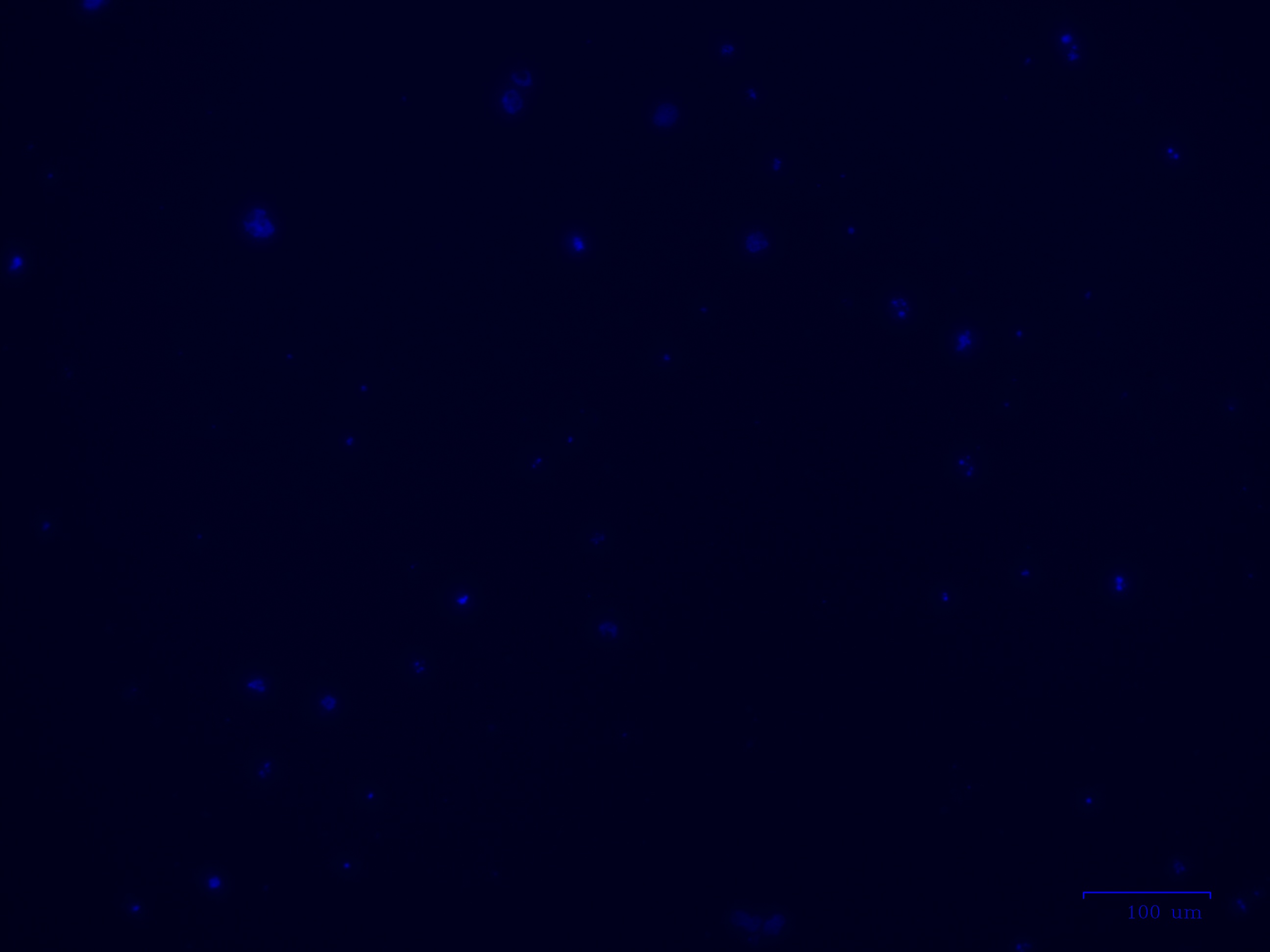

Supplement: Figure S4 — DAPI staining of WT iPSCs treated with 1 uM Digoxin [file peerj-08-9060-s006.jpg]

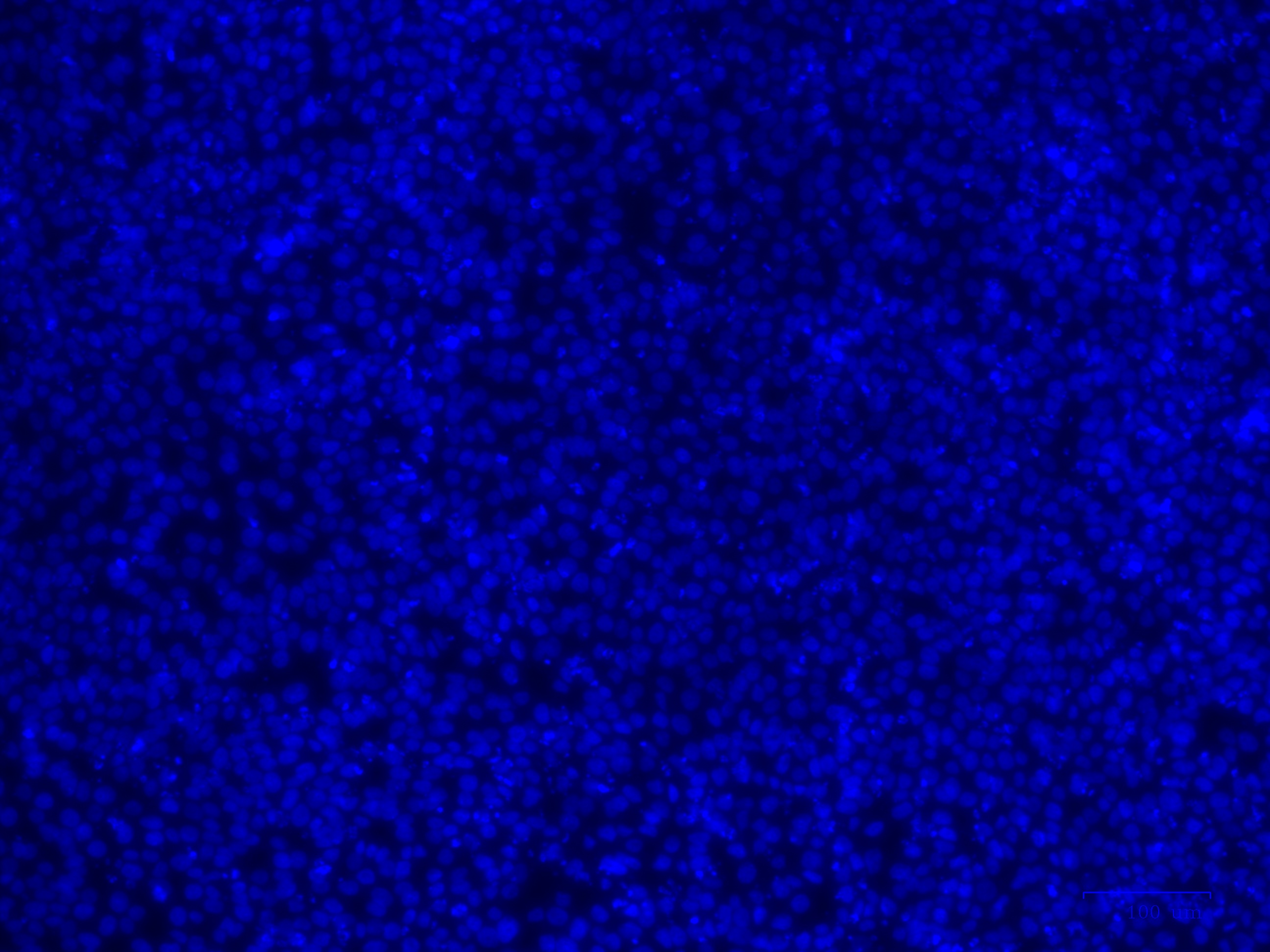

Supplement: Figure S5 — DAPI staining of ATP1A1 (118R/129D) iPSCs treated with 1 uM Digoxin [file peerj-08-9060-s007.jpg]

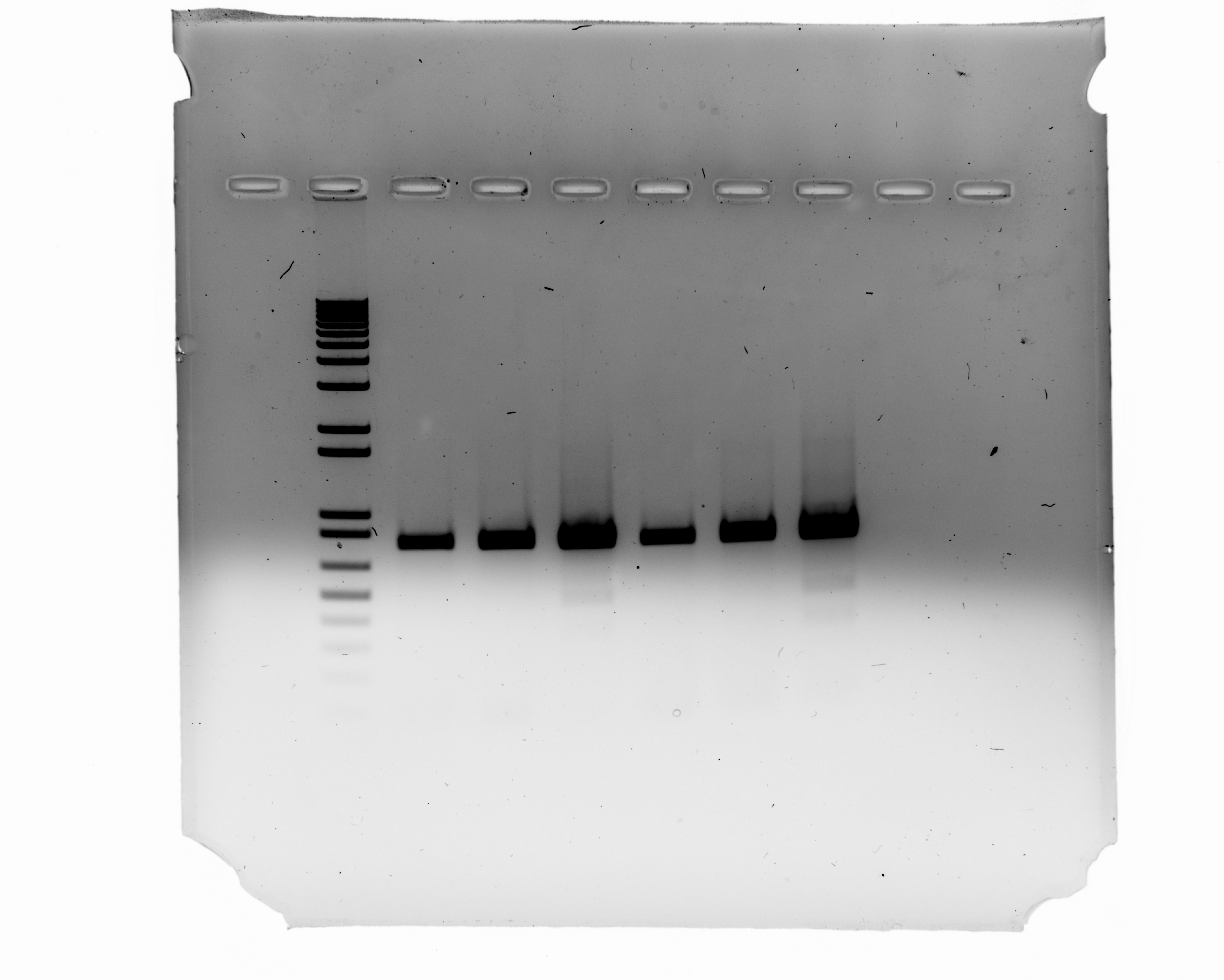

Supplement: Figure S6 — Lane 1:1kb ladder; Lane2: K3 iPSC spCas9(-), Donor(-), Digoxin (-); Lane3: K3 iPSC spCas9(+), Donor(+), Digoxin(-); Lane4: K3 iPSC spCas9(+), Donor(+), Digoxin(+); Lane5: SV20 iPSC spCas9(-), Donor(-), Digoxin(-); Lane6: SV20 iPSC spCas9(+), Donor(+), Digoxin(-); Lane7: SV20 iPSC spCas9(+), Donor(+), Digoxin(+) [file peerj-08-9060-s008.jpg]
